# Nomogram model of functional outcome for endovascular treatment in patients with acute basilar artery occlusion

**DOI:** 10.3389/fneur.2023.1277189

**Published:** 2023-10-19

**Authors:** Lei Li, Jin Lv, Jian-jia Han, Yuan Gao, Zhao-xuan Yan, Qi Wu, Xiao-li Zhang, Feng Gao

**Affiliations:** ^1^Interventional Neuroradiology, Department of Neurology, Beijing Tiantan Hospital, Capital Medical University, Beijing, China; ^2^Department of Radiotherapy, The PLA Rocket Force Characteristic Medical Center, Beijing, China; ^3^China National Clinical Research Center for Neurological Diseases, Beijing Tiantan Hospital, Capital Medical University, Beijing, China

**Keywords:** acute basilar artery occlusion, nomogram, endovascular treatment, favorable outcome, large vessel occlusion

## Abstract

**Background and purpose:**

The efficacy and safety of endovascular treatment (EVT) in acute basilar artery occlusion (ABAO) has been confirmed by four randomized clinical trials. Nevertheless, the predictors of a 90-day favorable outcome after EVT have not been elucidated. We attempted to establish a nomogram for the prediction of a 90-day favorable outcome in ABAO patients with EVT.

**Methods:**

Clinical data of ABAO patients with EVT were obtained from two nationwide clinical trial registries in China. Factors associated with a 90-day favorable outcome were screened by multivariable step-wise regression on the basis of univariable analysis. A nomogram was established to predict 90-day favorable outcome after EVT.

**Results:**

The proportion of ABAO patients with a favorable outcome was 41.53% (157/378). Seven variables, including baseline National Institutes of Health Stroke Scale (NIHSS) <20 [odds ratio (OR): 8.330; *P*-value < 0.0001], posterior circulation Alberta Stroke Program Early CT (pc-ASPECT) score ≥7 (OR: 1.948; *P*-value = 0.0296), Pons-Midbrain Index (PMI) score < 2 (OR: 2.108; *P*-value = 0.0128), Posterior Circulation Collateral Score (PC-CS) ≥5 (OR: 3.288; *P*-value < 0.0001), local anesthesia (OR: 0.389; *P*-value = 0.0017), time from onset to recanalization (OTR) <330 min (OR: 2.594; *P*-value = 0.0013), and no occurrence of early neurological deterioration (END; OR: 0.039; *P*-value < 0.0001) were included into the nomogram, with C-index values of 0.8730 and 0.8857 in the training and the internal validation set, respectively.

**Conclusions:**

The proposed nomogram provided a reliable prognostic scale, which can be employed in clinical settings for the selection and clinical management of ABAO patients.

**Registration:**

https://www.clinicaltrials.gov, identifier: NCT03370939.

## 1. Introduction

The clinical benefits of endovascular treatment (EVT) for acute ischemic stroke (AIS) patients with intracranial large artery occlusions (LVOs) in the anterior circulation, whether within or beyond the time window, have been confirmed in seven randomized clinical trials (RCTs) (1–7). The previous Basilar Artery Occlusion Endovascular Intervention Versus Standard Medical Treatment (BEST) (8) and Basilar Artery International Cooperation Study (BASICS) (9) clinical trials failed to demonstrate the superior effectiveness of EVT over standard medical therapies in acute basilar artery occlusion (ABAO). Nevertheless, these two randomized trials confirmed the safety of EVT in ABAO. The subsequent Basilar Artery Occlusion Chinese Endovascular Trial (BAOCHE) (10) and the Endovascular Treatment for Acute Basilar Artery Occlusion (ATTENTION) (11) randomized clinical trials decisively demonstrated the beneficial effects of EVT in ABAO patients within 24 h of symptom onset, of which, the rate of favorable outcome is consistent with previous observations in patients with anterior circulation large vessel occlusion (AC-LVO) ischemic stroke. Compared to the insufficient imaging-related parameters in patient selection for EVT in anterior circulation strokes, a more rigorous imaging selection of patients was employed in EVT on ABAO patients, which might contribute to the added benefit of EVT in posterior circulation strokes. Besides the imaging parameters such as the posterior circulation Acute Stroke Prognosis Early CT Scores (pc-ASPECTS) and collateral status, the occlusion site, the time period between the onset and the treatment, and the severity of the initial infarction were also identified to be the predictors of the outcomes of EVT for ABAO patients (12–17). However, most of the studies that included these parameters to predict the functional outcome of EVT were obtained from small samples or retrospective studies (18–20). Being a simple statistical visual tool, the nomogram has been used to predict the incidence of complication, prognostic recovery and mortality after EVT of AC-LVO (21–23). Nevertheless, nomograms for predicting clinically favorable outcomes of EVT in patients with posterior circulation large vessel occlusion (PC-LVO) are scarce.

In this study, we established a nomogram to identify the preprocedural and peri-procedural factors for EVT outcome in ABAO patients that enable the prediction of functional outcome and assist clinicians in patient selection and clinical management.

## 2. Materials and methods

### 2.1. Data availability

For purposes of replicating the procedure or reproducing the results, data are available to researchers upon request, by directly contacting the corresponding author.

### 2.2. Study design and population

This study is a retrospective analysis of the prospective data from the Acute Ischemic Stroke Cooperation Group of Endovascular Treatment (ANGEL) (24) and the Endovascular Treatment Key Technique and Emergency Work Flow Improvement of Acute Ischemic Stroke (ANGEL-ACT) (25) clinical trial registries. Details regarding the design of these clinical trial studies, the criteria for patient inclusion/exclusion, and standards for data collection, have been discussed in previous publications. The ethics committees of all participating centers approved these study protocols, and written informed consent was provided by the study participants or their legal representatives. This study was performed in compliance with the 1964 Declaration of Helsinki and its later amendments. Of the 2,710 patients included in these registries, 2,299 were excluded from the present analysis, due to anterior circulation occlusion (*n* = 2,071), vertebral artery occlusion (*n* = 174), or posterior cerebral artery occlusion (*n* = 54). The remaining 411 ABAO patients were further excluded if they have not undergone angiographic collateral assessment (*n* = 25), or a 90-day follow-up stage (*n* = 8). In total, 378 patients were retained in the cohort for subsequent analyses.

### 2.3. Data collection

The prospectively collected variables included age, sex, baseline modified Rankin Scale (mRS) score, baseline National Institutes of Health Stroke Scale (NIHSS) score, intravenous thrombolysis, mechanical thrombectomy (MT) procedure details, and follow-up outcomes. Neuroimaging variables included the posterior circulation Alberta Stroke Program Early CT Score [pc-ASPECTS] (26), the posterior circulation collateral score (PC-CS) (27), and the Basilar Artery on Computed Tomography Angiography (BATMAN) score (28).

### 2.4. Outcome definitions

Functional outcomes in this study included a favorable outcome [modified Rankin Scale (mRS) of 0–2] and patient mortality within 90 days. Early neurological deterioration (END) was defined as an increase of the NIHSS score of ≥4 points compared to the baseline NIHSS, or if the patient died within 24 h after EVT. Successful recanalization was defined as a modified thrombolysis in a cerebral infarction grade (mTICI) score of 2b or 3, while futile recanalization was defined as 90-day mRS score of 3–6, despite a technically successful recanalization (mTICI score of 2b or 3). Symptomatic intracranial hemorrhage (sICH) was defined as a hemorrhage detected by CT/MRI, with an increase of ≥4 points or an increase of ≥2 points of the NIHSS score in any NIHSS domain compared with the immediate prehemorrhage neurological status, or the need for surgical treatment according to Heidelberg Bleeding Classification criteria (29). Trained investigators, who were blinded to patient baseline conditions, conducted 90-day follow-up outcome assessments via telephone interviews, using a standard interview protocol.

### 2.5. Statistical analysis

All statistical analyses were performed using SAS version 9.4 (SAS Institute, Cary, NC, USA) and R version 4.2.1 software (http://www.R-project.org, foundation for statistical computing, Vienna, Austria). For continuous variables, data are presented as the median and interquartile ranges (IQRs). For categorical variables, data are presented as frequency and percentage. The optimal cutoff value for each continuous variable in predicting the probability of favorable outcome after EVT in ABAO patients was calculated by a receiver operating characteristics (ROC) curve, in which all continuous variables were converted into categorical variables. Univariable logistic regression analysis was performed to compare variables in the framework of demographic, medical history, etiology, imaging parameters, procedural parameters, times and incidence of complication, between the favorable outcome and the unfavorable outcome groups. To test for collinearity between predictors of the multivariable logistic model, the variance inflation factor (VIF) of each predictor was calculated. No predictors with a VIF >5 were included in the final regression model (30). All statistical tests were two-tailed and a *P-*value < 0.05 indicated a statistically significant difference, and variables with a *P-*value < 0.05 in the univariable analysis were included into a step-wise regression model. Furthermore, a nomogram was constructed for the prediction of a 90-day favorable outcome in ABAO patients undergoing EVT, by assigning a preliminary score to each predictor, ranging from 0 to 100 points. The total scores were obtained by summing the individual scores of each predictor. Then an internal validation step was performed by bootstrap resampling with 1,000 replications, according to an 8:2 ratio from the whole training set (302 cases). The discriminative performance of the nomogram model was assessed by a concordance index value, with values ranging between 0.5 (for a noninformative model) and 1 (for a perfectly discriminating model). The calibration plot was used to evaluate the degree of fit between the actual and the nomogram-predicted favorable outcomes. Decision curve analysis was used to discriminate the net benefit and the probability of a favorable outcome at 90 days, for ABAO patients undergoing EVT.

## 3. Results

### 3.1. General characteristics of the participants

**Figure 1:**
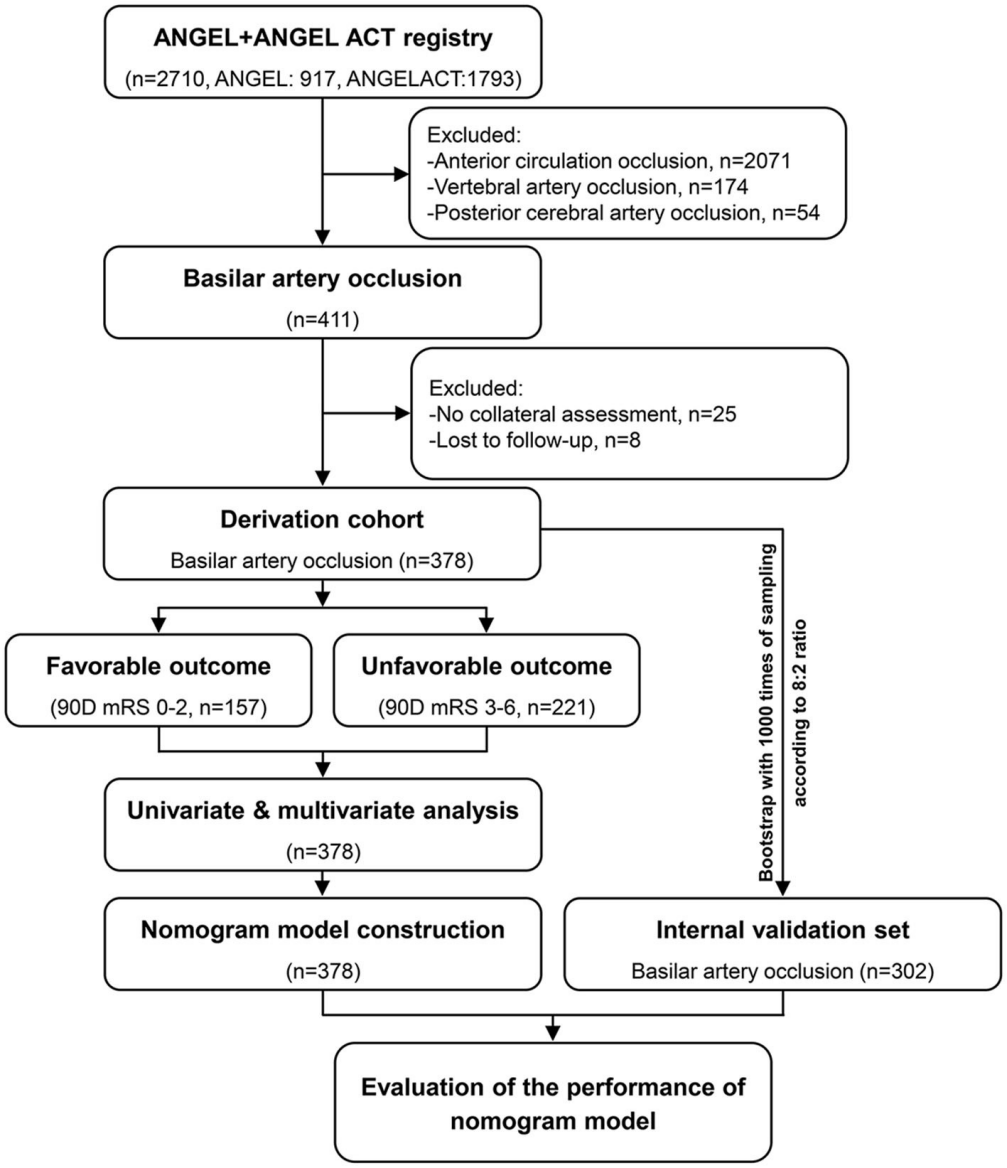
Flow chart of eligible ABAO patients with EVT, obtained from the ANGEL and ANGEL-ACT clinical trial registries. ABAO, acute basilar artery occlusion; EVT, endovascular treatment; ANGEL, Acute Ischemic Stroke Cooperation Group of Endovascular Treatment; ANGEL-ACT, Endovascular Treatment Key Technique and Emergency Work Flow Improvement of Acute Ischemic Stroke.

**Figure 2:**
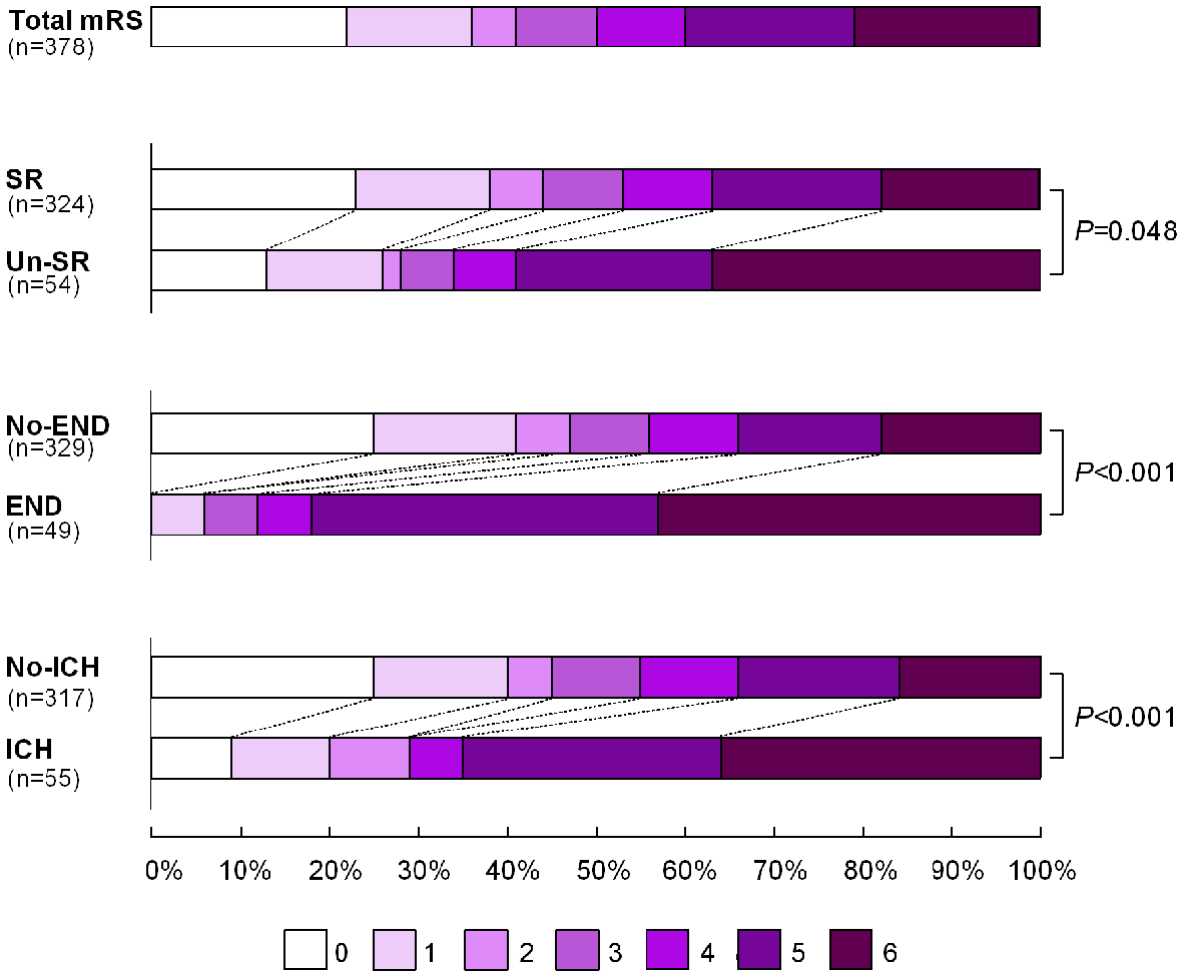
The distribution of the modified Rankin Scale (mRS) of patients with SR vs. Un-SR; END vs. no-END; and ICH vs. no-ICH. The mRS scores ranged from 0 to 6, with the higher scores indicating a more severe patient disability. SR, successful recanalization; Un-SR, un-successful recanalization; END, early neurological deterioration; ICH, intracerebral hemorrhage.

In the training set, a total of 115 patients in the ANGEL and 296 patients in the ANGEL-ACT registries were included. Thirty-three patients were excluded for missing collateral assessment data (*n* = 25), or due to the absence of follow-up information (*n* = 8). Finally, 378 basilar artery occlusion patients who underwent EVT were included in the final analyses (Figure 1). The mean age of the ABAO patients who underwent EVT was 62 years (IQR: 55–68), and 81.75% (309/378) of the included patients were male. The median baseline NIHSS score was 20 (IQR: 10–33), the median pc-ASPECT score was 7 (IQR: 6–8), the median PMI was 2 (IQR: 0–3), the median PC-CS score was 4 (IQR: 3–6), and the median BATMAN score was 4 (IQR: 4–6). The median time from symptom onset to puncture was 360 min (IQR: 240–540). 22.22% (84/378) patients received intravenous recombinant tissue plasminogen activator (IV-rtPA). The rate of successful recanalization was 85.71% (324/378), of which 56.17% (182/324) of the patients still suffered from futile recanalization. Concerning complications within 24 h, END was observed in 12.96% (49/378) ABAO patients post-EVT, ICH occurred in 14.78% (55/378) ABAO patients, and 4.05% (15/378) patients developed sICH after EVT. The 90-day mortality rate was 20.90% (79/378). A favorable outcome was found in 41.53% (157/378) of ABAO patients during the 90 days follow-up. The general characteristics and procedure parameters are listed in Supplementary Table 1. The modified Rankin Scale (mRS) scores distribution of patients with successful recanalization vs. un-successful recanalization, the END vs. no-END, and the ICH vs. no-ICH were compared and are shown in Figure 2.

### 3.2. Predictors of a 90-day favorable outcome in ABAO patients undergoing endovascular treatment

The baseline characteristics of the ABAO patients in the training and in the internal validation set, grouped by 90-day favorable outcome (mRS 0–2 vs. 3–6), are listed in Table 1. The continuous variables were converted into dichotomized variables by the optimal cutoff identified by receiver operating curve analysis in differentiating the favorable outcome. The detailed information is listed in Supplementary Table 2. By using a univariable analysis, the baseline NIHSS score, four neuroimaging variables including pc-ASPECT, PMI, PC-CS, and BATMAN, the anesthesia method, the final mTICI, OTP, END, and 24 h ICH were identified as potential predictors (*P*-values < 0.05). After screening by step-wise logistic regression method, a baseline NIHSS < 20 [OR: 8.330 (95% CI: 4.661–14.888); *P*-value < 0.0001], PC-ASPECT score ≥7 [OR: 1.948 (95% CI: 1.068–3.554); *P*-value = 0.0296], PMI score < 2 [OR: 2.108 (95% CI: 1.172–3.792); *P*-value = 0.0128], PC-CS score ≥5 [OR: 3.288 (95% CI: 1.876–5.760); *P*-value < 0.0001], local anesthesia method [OR: 0.389 (95% CI: 0.215–0.701); *P*-value = 0.0017], time from symptom onset to puncture < 330 min [OR: 2.594 (95% CI: 1.453–4.629); *P*-value = 0.0013], and no occurrence of END [OR: 0.039 (95% CI: 0.011–0.142); *P*-value < 0.0001] were associated with the 90-day favorable outcome in ABAO patients with EVT (Table 2).

**Table 1:** Univariable analysis of the factor associated with favorable outcome (90D mRS ≤ 2) in acute basilar artery occlusion (ABAO) patients undergoing endovascular treatment (EVT).

**Variables**	**Statistics**	**Training set (*****n*** = **378)**		**Internal validation set (*****n*** = **302)**	
		**mRS ≤ 2 (*n* = 157)**	**mRS ≥3 (*n* = 221)**	**mRS ≤ 2 (*n* = 131)**	**mRS ≥3 (*n* = 171)**
**Demographics**					
Age	Median, IQR	62 IQR (54~68)	62 IQR (55~69)	61 IQR (52~67)	63 IQR (56~69)
	≥68	26.11% (41/157)	29.41% (65/221)	24.43% (32/131)	30.41% (52/171)
	< 68	73.89% (116/157)	70.59% (156/221)	75.57% (99/131)	69.59% (119/171)
	*P*-value	0.5614		0.3001	
Gender	Male	82.80% (130/157)	81.00% (179/221)	84.73% (111/131)	78.36% (134/171)
	Female	17.20% (27/157)	19.00% (42/221)	15.27% (20/131)	21.64% (37/171)
	*P*-value	0.6871		0.1831	
**Medical history**					
Smoking habits	Yes	38.22% (60/157)	43.44% (96/221)	42.75% (56/131)	38.01% (65/171)
	No	42.68% (67/157)	37.10% (82/221)	38.93% (51/131)	42.69% (73/171)
	Quit	19.11% (30/157)	19.46% (43/221)	18.32% (24/131)	19.30% (33/171)
	*P*-value	0.513		0.7021	
Hypertension	Yes	71.34% (112/157)	71.49% (158/221)	73.28% (96/131)	72.51% (124/171)
	No	28.66% (45/157)	28.51% (63/221)	26.72% (35/131)	27.49% (47/171)
	*P*-value	1.0000		0.8969	
Diabetes mellitus	Yes	23.57% (37/157)	26.70% (59/221)	24.43% (32/131)	23.98% (41/171)
	No	76.43% (120/157)	73.30% (162/221)	75.57% (99/131)	76.02% (130/171)
	*P*-value	0.5493		1.0000	
Hyperlipemia	Yes	17.83% (28/157)	14.03% (31/221)	17.56% (23/131)	14.04% (24/171)
	No	82.17% (129/157)	85.97% (190/221)	82.44% (108/131)	85.96% (147/171)
	*P*-value	0.3184		0.4264	
Coronary heart disease	Yes	9.55% (15/157)	13.12% (29/221)	10.69% (14/131)	13.45% (23/171)
	No	90.45% (142/157)	86.88% (192/221)	89.31% (117/131)	86.55% (148/171)
	*P*-value	0.3307		0.4858	
Atrial fibrillation	Yes	9.55% (15/157)	9.05% (20/221)	9.16% (12/131)	9.94% (17/171)
	No	90.45% (142/157)	90.95% (201/221)	90.84% (119/131)	90.06% (154/171)
	*P*-value	0.8593		0.8468	
Previous stroke	Yes	23.57% (37/157)	24.43% (54/221)	23.66% (31/131)	22.81% (39/171)
	No	76.43% (120/157)	75.57% (167/221)	76.34% (100/131)	77.19% (132/171)
	*P*-value	0.9031		0.8911	
Pre mRS score	=0	90.45% (142/157)	90.05% (199/221)	9.92% (13/131)	8.19% (14/171)
	≥1	9.55% (15/157)	9.95% (22/221)	90.08% (118/131)	91.81% (157/171)
	*P*-value	1.0000		0.6853	
Pre-IVT	Yes	24.84% (39/157)	20.36% (45/221)	23.66% (31/131)	21.05% (36/171)
	No	75.16% (118/157)	79.64% (176/221)	76.34% (100/131)	78.95% (135/171)
	*P*-value	0.3172		0.6753	
**Etiology (TOAST)**					
	LAA	75.80% (119/157)	73.30% (162/221)	77.10% (101/131)	73.10% (125/171)
	CE	19.11% (30/157)	16.29% (36/221)	18.32% (24/131)	16.37% (28/171)
	SOE	2.55% (4/157)	3.62% (8/221)	2.29% (3/131)	3.51% (6/171)
	SUE	2.55% (4/157)	6.79% (15/221)	2.29% (3/131)	7.02% (12/171)
	*P*-value	0.2208		0.2261	
SBP	Median, IQR	157 IQR (140~175)	152 IQR (140~170)	158 IQR (142~175)	152 IQR (40~170)
	≥155	56.05% (88/157)	46.61% (103/221)	58.78% (77/131)	45.61% (78/171)
	< 155	43.95% (69/157)	53.39% (118/221)	41.22% (54/131)	54.39% (93/171)
	*P*-value	0.0765		0.0274	
DBP	Median, IQR	89 IQR (80~100)	90 IQR (80~97)	89 IQR (80~100)	89 IQR (80~95)
	≥84	68.15% (107/157)	60.73% (133/219)	69.47% (91/131)	59.17% (100/169)
	< 84	31.85% (50/157)	39.27% (86/219)	30.53% (40/131)	40.83% (69/169)
	*P*-value	0.1575		0.0706	
Baseline NIHSS	Median, IQR	12 IQR (6~20)	28 IQR (16~35)	12 IQR (6~21)	29 IQR (16~35)
	≥20	29.30% (46/157)	68.78% (152/221)	29.01% (38/131)	69.59% (119/171)
	< 20	70.70% (111/157)	31.22% (69/221)	70.99% (93/131)	30.41% (52/171)
	*P-*value	< 0.0001		< 0.0001	
**Neuroimaging variables**					
PC-ASPECT	Median, IQR	8 IQR (7~9)	6 IQR (5~8)	8 IQR (7~9)	6 IQR (5~8)
	≥7	75.80% (119/157)	49.32% (109/221)	75.57% (99/131)	49.71% (85/171)
	< 7	24.20% (38/157)	50.68% (112/221)	24.43% (32/131)	50.29% (86/171)
	*P*-value	< 0.0001		< 0.0001	
PMI	Median, IQR	1 IQR (0~2)	2 IQR (1~4)	1 IQR (0~2)	2 IQR (0~4)
	≥2	46.50% (73/157)	70.59% (156/221)	45.04% (59/131)	67.84% (116/171)
	< 2	53.50% (84/157)	29.41% (65/221)	54.96% (72/131)	32.16% (55/171)
	*P*-value	< 0.0001		< 0.0001	
PC-CS	Median, IQR	5 IQR (4~7)	4 IQR (3~5)	6 IQR (4~7)	4 IQR (3~5)
	≥5	64.97% (102/157)	33.94% (75/221)	66.41% (87/131)	34.50% (59/171)
	< 5	35.03% (55/157)	66.06% (146/221)	33.59% (44/131)	65.50% (112/171)
	*P*-value	< 0.0001		< 0.0001	
BATMAN	Median, IQR	5 IQR (3~7)	4 IQR (2~5)	5 IQR (3~7)	4 IQR (2~5)
	≥6	40.76% (64/157)	19.91% (44/221)	43.51% (57/131)	21.05% (36/171)
	< 6	59.24% (93/157)	80.09% (177/221)	56.49% (74/131)	78.95% (135/171)
	*P*-value	< 0.0001		< 0.0001	
**Procedures**					
Anesthesia	GA	56.69% (89/157)	76.02% (168/221)	55.73% (73/131)	74.85% (128/171)
	Local	43.31% (68/157)	23.98% (53/221)	44.27% (58/131)	25.15% (43/171)
	*P*-value	< 0.0001		0.0006	
Heparin	Yes	43.31% (68/157)	46.61% (103/221)	45.04% (59/131)	44.44% (76/171)
	No	56.69% (89/157)	53.39% (118/221)	54.96% (72/131)	55.56% (95/171)
	*P*-value	0.5314		1.0000	
Antagonist (GP2b3a)	Yes	70.06% (110/157)	70.59% (156/221)	71.76% (94/131)	72.51% (124/171)
	No	29.94% (47/157)	29.41% (65/221)	28.24% (37/131)	27.49% (47/171)
	*P*-value	0.9096		0.8975	
Tandem	Yes	10.19% (16/157)	14.03% (31/221)	8.40% (11/131)	14.04% (24/171)
	No	89.81% (141/157)	85.97% (190/221)	91.6% (120/131)	85.96% (147/171)
	*P*-value	0.3427		0.1489	
Residual severe stenosis	Yes	57.32% (90/157)	59.73% (132/221)	59.54% (78/131)	60.82% (104/171)
	No	38.22% (60/157)	37.10% (82/221)	35.88% (47/131)	35.67% (61/171)
	Unknown	4.46% (7/157)	3.17% (7/221)	4.58% (6/131)	3.51% (6/171)
	*P*-value	0.7680		0.8899	
Numbers of thrombectomy	≤ 3	94.90% (149/157)	91.86% (203/221)	95.42% (125/131)	91.23% (156/171)
	>3	5.10% (8/157)	8.14% (18/221)	4.58% (6/131)	8.77% (15/171)
	*P*-value	0.3049		0.1772	
Number of aspirations	0	88.54% (139/157)	92.76% (205/221)	90.08% (118/131)	92.98% (159/171)
	1	8.28% (13/157)	4.98% (11/221)	7.63% (10/131)	4.09% (7/171)
	2	1.91% (3/157)	0.45% (1/221)	2.29% (3/131)	0.58% (1/171)
	3	1.27% (2/157)	0.45% (1/221)	0.00% (0/131)	0.58% (1/171)
	4	0.00% (0/157)	1.36% (3/221)	0.00% (0/131)	1.75% (3/171)
	*P*-value	0.1089		0.0953	
Number of balloon dilatation	0	63.69% (100/157)	51.13% (113/221)	64.12% (84/131)	48.54% (83/171)
	1	31.21% (49/157)	44.34% (98/221)	30.53% (40/131)	46.78% (80/171)
	≥2	5.10% (8/157)	4.52% (10/221)	5.34% (7/131)	4.68% (8/171)
	*P*-value	0.0337		0.0154	
Number of stent implantation	0	61.15% (96/157)	55.66% (123/221)	58.78% (77/131)	54.39% (93/171)
	1	36.31% (57/157)	42.53% (94/221)	38.17% (50/131)	45.03% (77/171)
	2	2.55% (4/157)	0.90% (2/221)	3.05% (4/131)	0.00% (0/171)
	3	0.00% (0/157)	0.90% (2/221)	0.00% (0/131)	0.58% (1/171)
	*P*-value	0.1674		0.0305	
Number of IA thrombolysis	0	83.44% (131/157)	82.81% (183/221)	86.26% (113/131)	81.87% (140/171)
	1	15.92% (25/157)	16.74% (37/221)	12.98% (17/131)	18.13% (31/171)
	2	0.64% (1/157)	0.45% (1/221)	0.76% (1/131)	0.00% (0/171)
	*P*-value	0.9511		0.2119	
Final mTICI	0 (0-2a)	9.55% (15/157)	17.65% (39/221)	7.63% (10/131)	16.96% (29/171)
	1 (2b-c)	90.45% (142/157)	82.35% (182/221)	92.37% (121/131)	83.04% (142/171)
	*P*-value	0.0360		0.0234	
Remote embolization	Yes	3.18% (5/157)	7.69% (17/221)	3.05% (4/131)	8.19% (14/171)
	No	96.82% (152/157)	92.31% (204/221)	96.95% (127/131)	91.81% (157/171)
	*P*-value	0.0760		0.0849	
Dissection	Yes	1.91% (3/157)	3.62% (8/221)	2.29% (3/131)	3.51% (6/171)
	No	98.09% (154/157)	96.38% (213/221)	97.71% (128/131)	96.49% (165/171)
	*P*-value	0.3737		0.7364	
**Times**					
OTA	Median, IQR	181 IQR (62~344)	261 IQR (120~427)	181 IQR (80~357)	270 IQR (130~437)
	≥314	26.11% (41/157)	40.27% (89/221)	25.95% (34/131)	39.77% (68/171)
	< 314	73.89% (116/157)	59.73% (132/221)	74.05% (97/131)	60.23% (103/171)
	*P*-value	0.0044		0.0140	
OTP	Median, IQR	310 IQR (208~495)	405 IQR (270~560)	330 IQR (194~495)	390 IQR (258~540)
	≥330	49.68% (78/157)	68.78% (152/221)	50.38% (66/131)	68.42% (117/171)
	< 330	50.32% (79/157)	31.22% (69/221)	49.62% (65/131)	31.58% (54/171)
	*P*-value	0.0003		0.0020	
PTR	Median, IQR	91 IQR (60~120)	111 IQR (60~156)	91 IQR (60~120)	111 IQR (60~154)
	≥139	17.20% (27/157)	31.67% (70/221)	16.03% (21/131)	30.41% (52/171)
	< 139	82.80% (130/157)	68.33% (151/221)	83.97% (110/131)	69.59% (119/171)
	*P*-value	0.0018		0.0043	
**Complications**					
END	Yes	1.91% (3/157)	20.81% (46/221)	0.76% (1/131)	19.88% (34/171)
	No	98.09% (154/157)	79.19% (175/221)	99.24% (130/131)	80.12% (137/171)
	*P*-value	< 0.0001		< 0.0001	
24ICH	Yes	10.19% (16/157)	18.14% (39/215)	11.45% (15/131)	21.08% (35/166)
	No	89.81% (141/157)	81.86% (176/215)	88.55% (116/131)	78.92% (131/166)
	*P*-value	0.0383		0.0295	
24h sICH	Yes	2.56% (4/156)	5.14% (11/214)	2.31% (3/130)	6.02% (10/166)
	No	97.44% (152/156)	94.86% (203/214)	97.69% (127/130)	93.98% (156/166)
	*P*-value	0.2887		0.1571	

LAA, large-artery atherosclerosis; CE, cardioembolic; SOE, stroke of other determined etiology; SUE, stroke of underdetermined etiology; SBP, systolic blood pressure; DBP, diastolic blood pressure; NIHSS, National Institute of Health Stroke Scale; mRS, modified Rankin scale; PC-ASPECT, posterior circulation Alberta Stroke Program Early CT Score; PMI, Pons-Midbrain Index; PC-CS, the posterior circulation collateral score; BATMAN, the Basilar Artery on Computed Tomography Angiography; mTICI, modified thrombolysis in cerebral infarction grade; OTA, onset to admission time; OTP, onset to puncture time; END, early neurological deterioration; PTR, puncture to reperfusion time; ICH, intracranial hemorrhage; sICH, symptomatic intracranial hemorrhage.

**Table 2:** Multivariate analysis of the predictors of 90D favorable outcome in acute basilar artery occlusion (ABAO) patients undergoing endovascular treatment (EVT).

**Variables**	**Training set (90D mRS of 0–2**, ***n*** = **157)**		**Validation set (90D mRS of 0–2**, ***n*** = **131)**	
	**OR (95% CI)**	***P*** **-value**	**OR (95% CI)**	***P*** **-value**
Baseline NIHSS (< 20)	8.330 (4.661–14.888)	< 0.0001	10.736 (5.568–20.701)	< 0.0001
PC-ASPECT (≥7)	1.948 (1.068–3.554)	0.0296		
PMI (< 2)	2.108 (1.172–3.792)	0.0128	2.656 (1.410–5.004)	0.0025
PC-CS (≥5)	3.288 (1.876–5.760)	< 0.0001	3.879 (2.044–7.364)	< 0.0001
Anesthesia method	0.389 (0.215–0.701)	0.0017	0.368 (0.190–0.712)	0.0030
OTP (< 330 min)	2.594 (1.453–4.629)	0.0013	2.828 (1.472–5.435)	0.0018
END	0.039 (0.011–0.142)	< 0.0001	0.013 (0.002–0.110)	< 0.0001

NIHSS, National Institute of Health Stroke Scale; PC-ASPECT, posterior circulation Alberta Stroke Program Early CT Score; PMI, Pons-Midbrain Index; PC-CS, the posterior circulation collateral score; OTP, onset to puncture time; END, early neurological deterioration.

### 3.3. Construction of a nomogram for the prediction of a 90-day favorable outcome in ABAO patients undergoing EVT and validation of its performance

**Figure 3:**
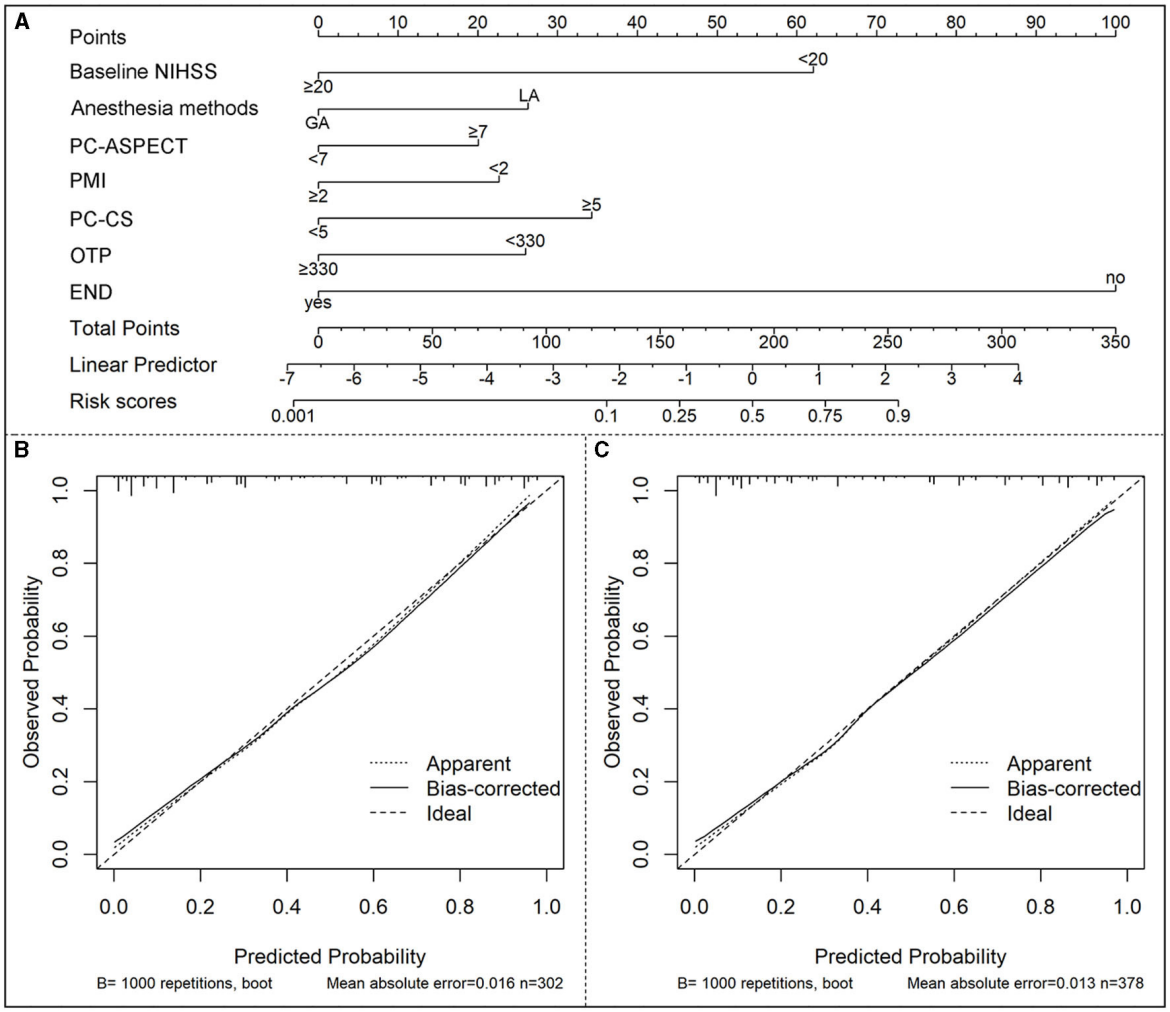
Nomogram model for the prediction of a 90-day favorable outcome (mRS of 0–2) in ABAO patients after EVT. **(A)** The nomogram developed in the present study; **(B)** Calibration curve of the training set. **(C)** Calibration curve of the internal validation set. ABAO, acute basilar artery occlusion; EVT, endovascular treatment; NIHSS indicates national Institute of Health Stroke Scale; PC-ASPECT, posterior circulation Alberta Stroke Program Early CT Score; PMI, Pons-Midbrain Index; PC-CS, the posterior circulation collateral score; OTP, onset to puncture time; END, early neurological deterioration.

**Figure 4:**
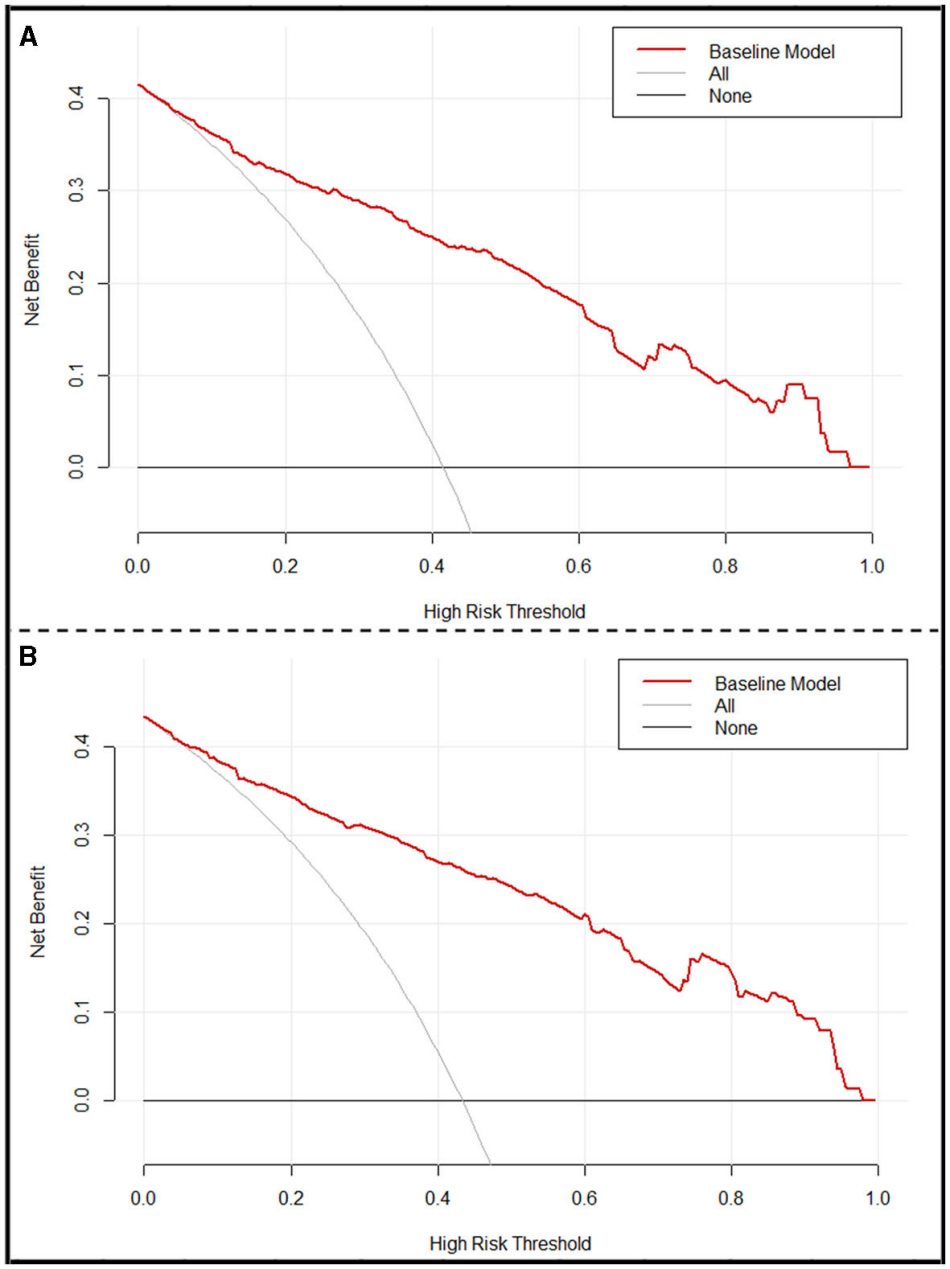
Decision curve analysis (DCA) of the nomogram. **(A)** DCA of the training set. **(B)** DCA of the internal validation set. *x*-axis, the threshold probability; *y*-axis, the net benefit. The gray line indicates that all ABAO patients undergoing EVT will achieve a favorable functional outcome 90 days after EVT. The black line indicates that no acute ischemic stroke patients undergoing EVT will obtain a favorable functional outcome 90 days after EVT. The red line corresponds to the nomogram to predict the 90-day favorable functional outcome in ABAO patients undergoing EVT. ABAO, acute basilar artery occlusion; EVT, endovascular treatment.

Based on the multivariable logistic regression model, a nomogram was developed to predict the probability of a 90-day favorable outcome, by assigning each independent predictor with a score ranging from 0 to 100 points. The cumulative sum of the assigned points for each factor in the nomogram represented the probability of a 90-day favorable outcome and ranged from 0 to 350 points. The probability of a 90-day favorable outcome ranged between 0.1 and 0.9 (Figure 3A). In order to evaluate the performance of the prognostic nomogram model, we carried out an internal validation for the nomogram (*n* = 302). The results of the corresponding univariable and step-wise multivariable logistic regression analysis of the internal validation set are listed in Table 2. The performance of the nomogram in the training and in the internal validation sets was evaluated by C-index, with results of 0.873 (95% CI: 0.838–0.908) and 0.886 (95% CI: 0.849–0.923), respectively. A calibration plot was further adjusted to assess the comparison between key summary features of the predictive factor scores and the agreement of a 90-day favorable outcome, between nomogram predictions and actual observations. These scores revealed a good predictive accuracy, both in the training and in the internal validation sets (Figures 3B, C). Finally, decision curve analysis was performed to evaluate the net benefit of the nomogram model in predicting a 90-day favorable outcome in ABAO patients after EVT. Figures 4A, B showed that when the risk threshold ranged from 1 to 96% (in the training set), and from 5 to 97%, (in internal validation sets), respectively, using the nomogram model to predict a 90-day favorable outcome resulted in a greater benefit than either all or none of the ABAO patients undergoing EVT. For example, if the personal threshold probability of a patient was 50%, then the net benefit in this nomogram model was 53.5% (95% CI: 40.7%−62.7%) in the training set and 55.7% (95% CI: 44.5%−66.2%) in the internal validation sets, respectively.

### 3.4. Translation of the nomogram model into clinical practice

**Figure 5:**
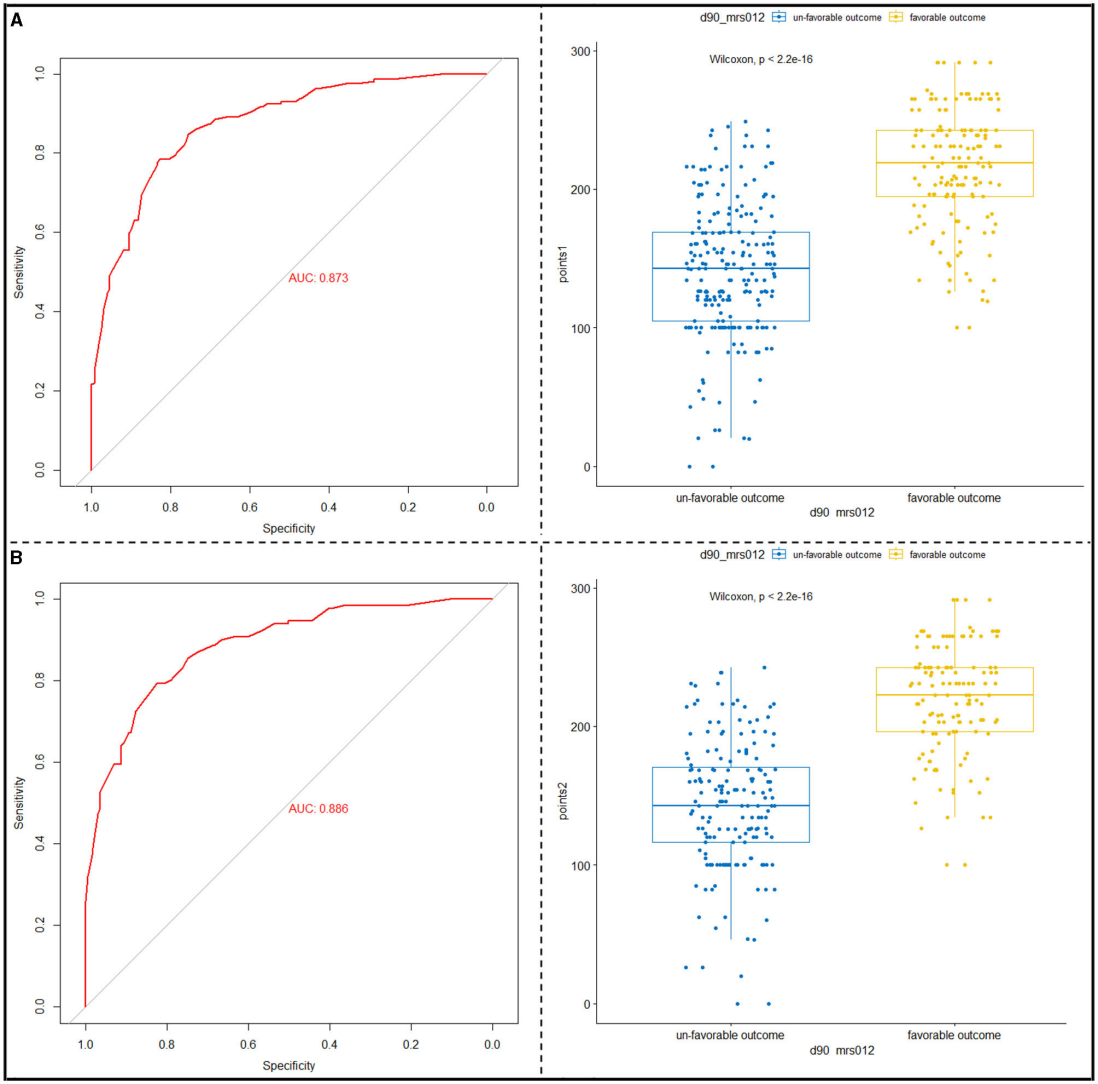
Translation of the nomogram model into clinical practice. **(A)** Receiver operating characteristics (ROC) for identifying the optimal threshold **(left)** and the scatter plot of the total scores **(right)** in ABAO patients undergoing EVT, in the training set. **(B)** ROC for identifying the optimal threshold **(left)** and scatter plot of the total scores **(right)** in ABAO patients undergoing EVT, in the internal validation set. ABAO, acute basilar artery occlusion; EVT, endovascular treatment.

Finally, we translated the predictive model into practice, by assigning the corresponding scores to each indicator, according to their contributions to the nomogram model, with 62 points for Baseline NIHSS scores < 20, 20 points for PC-ASPECT scores ≥7, 22.68 points for PMI scores < 2, 34.28 points for PC-CS scores ≥5, 52.57 points for the local anesthesia method and 25.97 points for OTP < 330 min, and 100 points for no-END. And the total scores of each patient were calculated as follows: total scores = (0 for NIHSS ≥20 or 1 for NIHSS < 20) × 62 + (0 for PC-ASPECT scores < 7 or 1 for PC-ASPECT scores ≥7) × 20 + (0 for PMI scores ≥2 or 1 for PMI scores < 2) × 22.68 + (0 for PC-CS scores < 5 or 1 for PC-CS scores ≥5) × 34.28 + (0 for general anesthesia method or 1 for local anesthesia) × 52.57 + (0 for OTP ≥330 min or 1 for OTP < 330 min) × 25.97 + (0 for END or 1 for no-END) × 100. The thresholds were 188.05 (in the training set) and 186.5 (in the internal validation set). Their sensitivities were 0.78344 (training set) and 0.82456 (internal validation set), and specificities were 0.82805 (training set) and 0.79389 (internal validation set). The probability of achieving functional independence was about 48%. The receiver operating curve and the scatter plot of the total scores of each patient in the training and in the internal validation sets are shown in Figures 5A, B.

## 4. Discussion

The present study introduces a nomogram model, which incorporated with both pre- and peri-procedural parameters, including baseline NIHSS, PC-ASPECT, PMI, PC-CS, the anesthesia method, OTP, and END, and with a good performance of C-index of 0.873, to predict a favorable outcome at 90 days after EVT in patients with ABAO.

**Figure 6:**
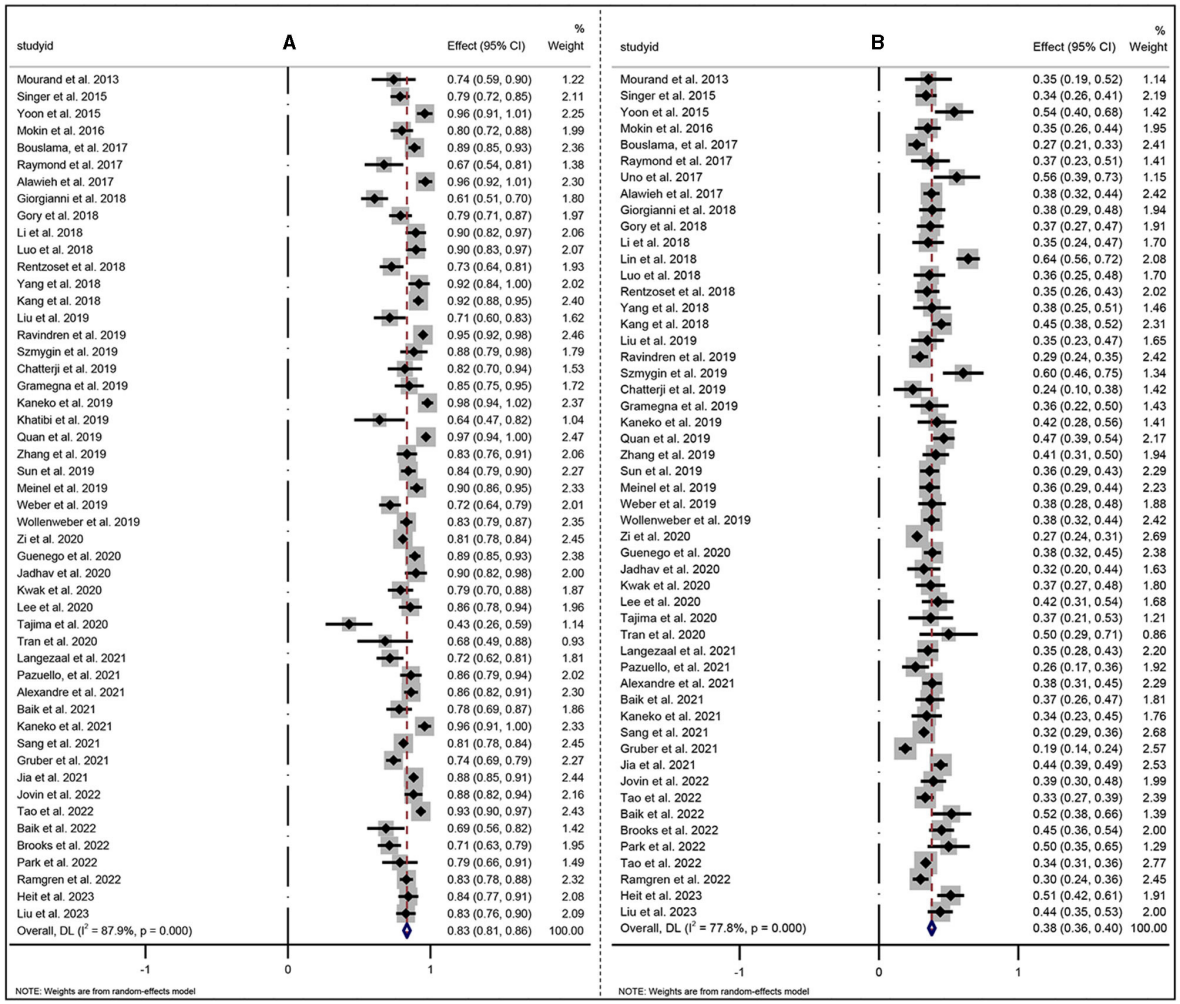
Forest plot of pooled incidence of successful recanalization **(A)** and 90-day favorable outcome (mRS of 0–2) **(B)** with random-effect methods in PC-LVO patients underwent EVT. PC-LVO, posterior circulation large vessel occlusion; EVT, endovascular treatment.

In this study, the successful reperfusion rate was 87.04% (329/378), which was slightly higher than the 71% rate in the HERMES (highly effective reperfusion evaluated in multiple endovascular stroke) (31) clinical trial, the 81% rate in the pooled 15 studies in a meta-analysis (32), and the 85% pooled rate in a meta-analysis (33) of four randomized clinical trials (RCTs), as well as the pooled 83% (95% CI: 81%−86%) rates of 50 studies that facilitated EVT on PC-LVO (Figure 6A) summarized in our study. The favorable outcome of this study was 41.5%, which was comparable to the 46% rate in the HEMERS analysis (31) and 42% reported by Gory et al. (32), and slightly higher than the pooled 35% rate in the meta-analysis (33) and the pooled 38% (95% CI: 36%−40%) rates of 52 studies (Figure 6B) summarized in our study. Given the relatively lower sICH rates of 4.05% and mortality rate of 20.9%, compared to those in the HERMES study (31), the meta-analysis of 15 studies (32) and the meta-analysis of four RCTs (33), our study also confirmed that EVT was safe for treating ABAO patients within 24 h of symptom onset.

The scoring system (34, 35) for predicting the prognosis and complications following EVT of AC-LVO have been extensively elucidated. Despite the benefit for MT reported by BOACHE (10) and ATTENTION (11) trials, the risk factors for functional outcome in ABAO patients after MT have not been elucidated. The POST-VB score (20) utilized post-procedure scans as the independent predictors of a 90-day favorable outcome, which did not specifically address patient selection. Besides, the generation of ASIAN KR Posterior Calculator (19) was substantially limited by its small sample size and the retrospective nature. To our knowledge, our model is the first predictive nomogram model incorporated with both pre- and peri-procedural factors for patient selection and clinical management of peri-operation in ABAO. Currently, the factors independently associated with a good outcome after EVT in PC-LVO (summarized in Supplementary Table 3) have considerably varied. Among which, baseline NIHSS was a well-recognized predictor associated with 90-day functional independence following the EVT of PC-LVO. Our study also found that a baseline NIHSS score < 20 was an independent predictor for a 90-day favorable outcome in ABAO patients, accounting for 17.7% (62/350) of total scores for the nomogram models. Rigorous imaging parameters have been confirmed to be an important selection criterion, which improved the substantial benefit of thrombectomy. Consistent with previous studies (13–15), we also identified that a PC-ASPECT score ≥7 was significantly correlated with a 90-day functional independence of ABAO patients who underwent EVT. The most recently published ATTENTION random trial (33) in the Chinese population has achieved an even higher successful recanalization rate of 93% in ABAO patients after EVT, which was largely attributed to the rigorous imaging selection of patients in the absence of a large baseline infarct. In addition to PC-ASPECT, our study found that patients with a PC-CS score ≥5 might achieve a more likely favorable outcome than those with a PC-CS score < 5 and a PMI score < 2 was an independent predictor of a favorable outcome in ABAO patients. Taken together, these findings strengthened the importance of imaging selection in predicting the prognosis of EVT in ABAO patients. The peri-procedural factors have been addressed in the studies of AC-LVO (21, 22), but few factors have been reported in PC-LVO studies. The higher incidence of END is often accompanied with a poor outcome, as well as by the increased disability and mortality following EVT in AC-LVO patients (36–40). Our study showed that no occurrences of END was associated with a favorable outcome in ABAO patients. Reducing END incidence is expected to ameliorate the prognosis. In agreement with the results from previous studies (19), we also found an association between the OTP and the clinical outcome in ABAO patients.

### 4.1. Limitation

The limitations of this study are as follows. Although an internal validation of the nomogram has been conducted in this study, a future external verification would be required to determine the generalizability of the model. In addition, the high proportion of intracranial atherosclerotic disease (ICAD) due to the different ethnicity of the population in this study and the low proportion of intravenous thrombolysis might affect the extrapolation of the study's findings. Nevertheless, there might be additional unknown factors that were not included in the model, which would require further extension studies.

## 5. Conclusion

Taken together, the proposed nomogram demonstrated a good discriminative performance in evaluating the probability of a 90-day favorable outcome for ABAO patients after EVT. This nomogram can be easily translated into practice, providing a reliable prognostic scale that can be employed in clinical settings, for the selection and clinical management of acute BAO patients.

## Data availability statement

The original contributions presented in the study are included in the article/Supplementary material, further inquiries can be directed to the corresponding author.

## Ethics statement

This study was approved by the Institutional Review Boards at Beijing Tiantan Hospital and at each trial site (KY2014-51-01 and 2017-048-01). The studies were conducted in accordance with the local legislation and institutional requirements. The participants provided their written informed consent to participate in this study.

## Author contributions

LL: Writing—original draft, Data curation, Investigation. JL: Writing—original draft, Conceptualization, Methodology, Writing—review and editing. J-jH: Conceptualization, Investigation, Writing—original draft. YG: Methodology, Writing—review and editing. Z-xY: Methodology, Writing—review and editing. QW: Investigation, Writing—review and editing. X-lZ: Data curation, Formal analysis, Methodology, Software, Writing—review and editing. FG: Conceptualization, Funding acquisition, Project administration, Resources, Writing—original draft, Writing—review and editing.
