# Supplementary material for: Nomogram model of functional outcome for endovascular treatment in patients with acute basilar artery occlusion
Source: Front Neurol. 2023 Oct 19;14:1277189. doi: 10.3389/fneur.2023.1277189 (PMC10621789; doi:10.3389/fneur.2023.1277189)
Supplement: Supplementary file 1 [file Data_Sheet_1.docx]

Supplementary Table 1 Baseline characteristics of acute basilar artery occlusion (ABAO) patients undergoing endovascular treatment (EVT).

| Variables | % (n); IQR (N=387) |
| --- | --- |
| Demographics |  |
| Age (years) (median, IQR) | 62 IQR (55～68) |
| ≥68 | 28.04% (106/378) |
| ＜68 | 71.96% (272/378) |
| Sex |  |
| Male | 81.75% (309/378) |
| Female | 18.25% (69/378) |
| Medical history |  |
| Smoke habits |  |
| Yes | 39.42% (149/378) |
| No | 41.27% (156/378) |
| Quit | 19.31% (/73/78) |
| Hypertension |  |
| Yes | 71.43% (270/378) |
| No | 28.57% (108/378) |
| Diabetes mellitus |  |
| Yes | 25.4% (96/378) |
| No | 74.6% (282/378) |
| Coronary heart disease |  |
| Yes | 11.64% (44/378) |
| No | 88.36 % (334/378) |
| Atrial fibrillation |  |
| Yes | 9.26% (35/378) |
| No | 90.74% (343/378) |
| Previous Stroke |  |
| Yes | 24.07% (91/378) |
| No | 75.93% (287/378) |
| Etiology |  |
| Large artery atherosclerosis (LAA) | 74.34% (281/378) |
| Cardioembolic (CE) | 17.46% (66/378) |
| Stroke of other determined etiology (SOE) | 3.17% (12/378) |
| Stroke of undetermined etiology (SUE) | 5.03% (19/378) |
| Pre-Intravenous thrombolysis |  |
| Yes | 22.22% (84/378) |
| No | 77.78% (294/378) |
| SBP | 155 IQR (140～170) |
| Baseline NIHSS (median, IQR) | 20 IQR (10～33) |
| PC-scores |  |
| PC-ASPECT (median, IQR) | 7 IQR (6～8) |
| PMI (median, IQR) | 2 IQR (0～3) |
| PC-CS (median, IQR) | 4 IQR (3～6) |
| BATMAN (median, IQR) | 4 IQR (3～6) |
| Procedures |  |
| Anesthesia |  |
| General | 67.99% (257/378) |
| Local | 32.01% (121/378) |
| Residual severe stenosis |  |
| Yes | 58.73% (222/378) |
| No | 37.57% (142/378) |
| Unknown | 3.70% (14/378) |
| Tandem |  |
| Yes | 12.43% (47/378) |
| No | 87.57% (331/378) |
| Final mTICI |  |
| 0 (0-2a) | 14.29% (54/378) |
| 1 (2b-c) | 85.71% (324/378) |
| Remote embolization |  |
| Yes | 5.82% (22/378) |
| No | 94.18% (356/378) |
| Dissection |  |
| Yes | 2.91% (11/378) |
| No | 97.09% (367/378) |
| Times |  |
| Time from onset to admission (median, IQR) | 240 IQR (110～420) |
| Time from onset to puncture (median, IQR) | 360 IQR (240～540) |
| Time from puncture to reperfusion (median, IQR) | 104 IQR (60～139.8) |
| Complications |  |
| Early neurological deterioration |  |
| Yes | 12.96% (49/378) |
| No | 87.04% (329/378) |
| 24h ICH |  |
| Yes | 14.78% (55/378) |
| No | 85.22% (317/378) |
| 24h sICH |  |
| Yes | 4.05% (15/378) |
| No | 95.95% (355/378) |
| 90-D mortality |  |
| Yes | 20.90%% (79/378) |
| No | 79.10% (299/378) |

LAA, large-artery atherosclerosis; CE, cardioembolic; SOE, stroke of other determined etiology; SUE, stroke of underdetermined etiology; SBP, systolic blood pressure; DBP, diastolic blood pressure; mRS indicates modified Rankin Scale; NIHSS indicates national Institute of Health Stroke Scale; mRS, modified Rankin scale; PC-ASPECT, posterior circulation Alberta Stroke Program Early CT Score; PMI, Pons-Midbrain Index; PC-CS, the posterior circulation collateral score; BATMAN, the Basilar Artery on Computed Tomography Angiography; mTICI, modified thrombolysis in cerebral infarction grade; OTA, onset to admission time; OTP, onset to puncture time; END, early neurological deterioration; PTR, puncture to reperfusion time; ICH, intracranial hemorrhage; sICH, symptomatic intracranial hemorrhage.

Supplementary Table 2 Continuous variables at optimal cutoff for predicting favorable outcome (90D mRS of 0-2)

| Variables | Cutoff | Sensitivity | Specificity | Accuracy | PLR | NLR | PPV | NPV |
| --- | --- | --- | --- | --- | --- | --- | --- | --- |
| age | 68 | 0.79618 | 0.17149 | 0.4894 | 1.09289 | 0.75074 | 0.43706 | 0.65217 |
| SBP | 155 | 0.56051 | 0.53394 | 0.54497 | 1.20265 | 0.82311 | 0.46073 | 0.63102 |
| DBP | 84 | 0.68153 | 0.39269 | 0.51330 | 1.12222 | 0.81099 | 0.44583 | 0.63235 |
| Baseline NIHSS | 20 | 0.75159 | 0.65611 | 0.69577 | 2.18555 | 0.37861 | 0.60825 | 0.78804 |
| PC-ASPECT | 7 | 0.75796 | 0.50679 | 0.61111 | 1.53678 | 0.47759 | 0.52193 | 0.74667 |
| PMI | 2 | 0.85350 | 0.47964 | 0.63492 | 1.64021 | 0.30543 | 0.53815 | 0.82171 |
| PCCS | 5 | 0.64968 | 0.66063 | 0.65608 | 1.91439 | 0.53028 | 0.57627 | 0.72637 |
| BATMAN | 6 | 0.40764 | 0.80090 | 0.63757 | 2.04748 | 0.73961 | 0.59259 | 0.65556 |
| OTA | 314 | 0.73885 | 0.40271 | 0.54233 | 1.23702 | 0.64846 | 0.46774 | 0.68642 |
| OTP | 330 | 0.50318 | 0.68778 | 0.61111 | 1.61165 | 0.72234 | 0.53378 | 0.66087 |
| PTR | 139 | 0.82803 | 0.31674 | 0.52910 | 1.21188 | 0.54295 | 0.46263 | 0.72165 |

SBP, systolic blood pressure; DBP, diastolic blood pressure; mRS indicates modified Rankin Scale; NIHSS indicates national Institute of Health Stroke Scale; mRS, modified Rankin scale; PC-ASPECT, posterior circulation Alberta Stroke Program Early CT Score; PMI, Pons-Midbrain Index; PCCS, the posterior circulation collateral score; BATMAN, the Basilar Artery on Computed Tomography Angiography; OTA, onset to admission time; OTP, onset to puncture time; PTR, puncture to reperfusion time; PLR = positive likelihood ratio; NLR = negative likelihood ratio; PPV = positive predictive value; NPV = negative predictive value.

Supplementary Table 3 Summary of predictors for 90D outcome following endovascular treatment (EVT) on acute basilar artery occlusion (ABAO)

| **Sources**  **Author/year** | **Study type and period** | **Location** | **Time window** | **Sample size** | **SR** | **Outcome (90D mRS)** | **AUC** | **Type of model** | **Factors for 90-day outcome following EVT** |
| --- | --- | --- | --- | --- | --- | --- | --- | --- | --- |
| Singer et al. 2015. [1] | ENDOSTROKE Registry  NCT1399762  2011.01.01-2013.06 | BAO | 60%<6h;  17%>6h;  22%unknown | N=148 | 79%  (111/141) | 0-3: 42%(62/148)  0-2: 34%(50/148) | NA | NA | Lower NIHSS: OR 0.917[0.877-0.959], P<0.001;  MRI: OR 4.586[1.453-14.477], P=0.009;  ASINT/SIR grade, categorized: OR 2.120[1.107-4.058], P=0.023; |
| Yoon et al. 2015. [2] | Retrospective study  2010.12-2015-02 | BAO | ≤12 h | N=50 | 96%(48/50) | 0-2: 54%(27/50) | NA | NA | Baseline NIHSS (per 1-point increase): OR 0.820[0.709-0.949], *P*=0.008;  Pc-ASPECT (per 1-point increase): OR 1.854[1.012-3.397], *P*=0.045; |
| Mokin et al. 2016. [3] | Retrospective study.  2012.03-2015.07 | PC | ≤24 h | N=100 | 80%(80/100) | 0-2: 35%(35/100) | NA | NA | Time of treatment within 0-6 h of onset: *P*=0.011;  OTR time within 0-6h (90cases): *P*=0.0039;  TICI 2b/3: *P*=0.00085; |
| Bouslama, et al. 2017. [4] | Retrospective study  2005.09-2015.09 | BAO or VBO | OTP: 621(285-1120) min | N=214 | 87.6%(190/214) | 0-2: 26.6%(58/214) | NA | NA | Smoking: OR 2.61[1.23-5.56], *P*=0.013;  Low baseline NIHSS: OR 1.09[1.04-1.13], *P*<0.0001;  SR: OR 10.80[1.36-85.96], *P*=0.025; |
| Uno et al. 2017. [5] | Retrospective study.  2011.10-2016.09 | BAO | ≤8 h | N=34 | 100% | 0-2: 56%(19/34) | NA | NA | IV-tPA use: OR 15.445[2.53-156.35], *P*=0.007;  NIHSS score: OR 0.889[0.78-0.98], *P*=0.036; |
| Giorgianni et al. 2018. [6] | Retrospective study.  2010.01-2015.12 | BAO | ≤24 h | N=102 | 61%(62/102) | 0-2: 38.2%(39/102) | NA | NA | Age: OR 1.08[1.021-1.128], *P*=0.008;  NIHSS score: OR 1.12[1.009-1.128], *P*=0.02;  Total time: OR 1.01[1.002-1.017], *P*=0.008; |
| Li et al. 2018. [7] | Retrospective study.  2014.01-2016.12 | BAO | ≤24 h | N=68 | 89.7%(61/68) | 0-2: 35.3%(24/68)  0-3: 45.6%(31/68) | NA | NA | Factor for favorable outcome(90D mRS 0-3)  Lower NIHSS: OR 0.832[0.715-0.968, *P*=0.018; |
| Lin et al. 2018. [8] | Retrospective study.  2015.01-2016.12 | PC | ≤24 h | N=125 |  | 0-2: 64%(80/125) | 0.7658 | NA | Predictor for unfavorable outcome  Age(>70 vs.<70): OR 2.84[1.14-7.09], *P*=0.0250;  Baseline NIHSS (per score change): OR 1.36[1.13-1.64], *P*=0.0013;  Pc-ASPECT(≤7vs.>7): OR 8.49[1.85-39.09], *P*=0.0060; |
| Luo et al. 2018. [9] | Retrospective study  2012.03-2016.11 | PC (V4, BA,or VA) | ≤12 h | N=69 | 89.9%(62/69) | 0-2: 36.2%(25/69) | NA | NA | Stroke subtype (ICAD vs Embolism): OR 0.101[0.020-0.501], *P*=0.005;  NIHSS on admission (≥22 vs. ＜22): OR 0.157[0.040-0.614], *P*=0.008;  Pc-ASPECT≥6vs.＜6: OR 7.355[1.495-36.191], *P*=0.014; |
| Rentzos et al. 2018. [10] | Retrospective study.  1991-2015. | PC | ≤12 h | N=110 | 73%(80/110) | 0-2: 35%(38/110) | NA | NA | BATMAN: OR 1.73[1.15-2.59], *P*=0.008;  Successful recanalization: OR 10.92[1.21-98.44], *P*=0.03; |
| Yang et al. 2018. [11] | Retrospective study.  2012.03-2016.12 | BAO | ≤24 h | N=50 | 92%(46/50) | 0-2: 38%(19/50) | 0.864[0.738-0.945] | NA | DWI BSS ≤2: OR 12.416[2.520-61.179], *P*=0.002; |
| Kang et al. 2018. [12] | Retrospective study termed ENTHUS  2011.01-2017.08 | BAO | ≤12 h | N=212 | 91.5%(194/212) | 0-2: 44.8%(95/212) | NA | NA | For better outcome: no predictor in multivariate analysis  For ordinal logistic regression model:  Younger age: OR 0.951[0.929-0.974], *P*<0.001;  Lower baseline NIHSS: OR 0.904[0.875-0.935]. *P*<0.001;  Absence of DM: OR 0.428[0.244-0.751], *P*=0.003;  Absence of PH: OR 0.074[0.014-0.312, *P*<0.001; |
| Ravindren et al. 2019. [13] | Retrospectively study.  2008.11-2019.02 | BAO | ≥6 h  OTR: 6.4h(2.5-20.4) | N=231 | 94.8%(219/231) | 0-2: 29.4%(68/231) | NA | NA | Time to recanalization (＞6h vs.≤6h), OR 0.47[0.23-0.96], *P*=0.036;  Year of treatment (from 2011vs. 2008-2010): OR 3.95[1.14-13.66], *P*=0.030;  Post image hemorrhage: OR 0.28[0.08-0.98], *P*=0.046; |
| Gramegna et al. 2019. [14] | Retrospectively study.  2015.01-2017.12 | PC | OTP: 290(189-464) min | N=47 | 85%(40/47) | 0-2: 36.2%(17/47) | NA | NA | Age<70 years: OR 6.20[1.52-25.47], *P*=0.01;  Absence of intracranial atherosclerosis: OR 6.45[1.09-38.24], *P*=0.04; |
| Kaneko et al. 2019. [15] | Retrospectively study.  2015.01-2017.12 | BAO | 281(216-617)  340(260-575)min | N=48 | 97.9%(47/48) | 0-3: 54.2%(26/48)  0-2: 41.7%(20/48) | NA | NA | Age: OR 1.14[1.03-1.27], *P*=0.01;  Pc-ASPECT: OR 0.45[0.24-0.85], *P*=0.01;  mTICI: OR 16.2[1.24-1.43], *P*=0.03; |
| Zhang et al. 2019. [16] | Retrospective study.  2012.04-2018.02 | VBO with ICAS | ＜24h | N=103 | 83.5% (86/103) | 0-2: 40.8%(42/103) | NA | NA | IVT: OR 7.343[1.621-33.263], *P*=0.010;  Prior antiplatelet: OR 7.301[1.761-30.265], *P*=0.006;  Pc-ASPECT(per 1-point increase): OR 1.705[1.127-2.580], *P*=0.012;  BATMAN: OR 1.395[1.005-1.937], *P*=0.047;  OTR(≤542 min vs. ＞542 min): OR 0.194[0.057-0.661], *P*=0.009;  NIHSS pretreatment (per 1-point increase): OR 0.882[0.820-0.949], *P*=0.001; |
| Guenego et al. 2021. [17] | Retrospective study. ETIS registry  2012.01-2019.03 | BAO | OTP: 316(226-458)min | N=237 | 89%(211/237) | 0-2: 38%(91/237) | NA | NA | Predictor for 90-D mRS 0-3:  Age: OR 0.95[0.91-0.98], *P*=0.007;  Baseline NIHSS: OR 0.87[0.82-0.92], *P*<0.0001;  ENI(early neurological improvement): OR 18.12[3.95-83.10], *P*=0.0001; |
| Jadhav et al. 2020. [18] | Prospective study.  2000.07-2012.07 | BAO | OTP: 13h (35) | N=59 | 90%(53/59) | 0-2: 32%(19/59) | AUC=0.91[0.84-0.98]  V1=0.89  V2=0.78  V3=0.80 | POST-VB score:  Age+(10×brainstem infarct volume) | Younger age: OR 1.04[1.01-1.07], *P*=;  Smaller brainstem infarct volume: OR 6.57[1.76-24.5], *P*<0.0001; |
| Kwak et al. 2020. [19] | Retrospective study 2012.01-2019.10 | BAO | OTR: 325(223-537.5) min | N=81 | 79.0%(64/81) | 0-2: 37.0%(30/81) | NA | NA | Baseline NIHSS ＜15: OR 8.49[2.01-35.82], *P*=0.004;  PCCS≥6: OR 3.79[1.05-13.66], *P*=0.042;  Distal BAO: OR 3.67[1.10-12.26], *P*=0.035; |
| Lee et al. 2020. [20] | Retrospective study on ASIAN KR registry  2011.01-2016.02  V: 2016.04-2018.12 | VBO | ≤24 h | Derivation set=71  Validation set=32 | D: 85.9%(61/71)  V: 78.1%(25/32) | 0-2  42.3%(30/71) | C(D)=0.86  C(V)=0.78 | Score calculator:  3.0×(DWIimaging infarct volume<10mL)  +2.2×(OTP<8h)  +1.8×(BSO).  ( 0 if no, 1 if yes) | DWI imaging volume≤10mL: OR 19.3 [3.0-126.4], *P*=0.002;  Onset to puncture time≤8h: OR 8.7 [1.8-42.0], *P*=0.007;  BSO(Branching-site occlusion): OR 6.1 [1.5-26.0], *P*=0.01; |
| Tajima et al. 2020. [21] | Retrospective analysis.  2014.07-2019.11 | BAO | ≤24 h | N=35 | TICI 3: 42.9%(15/35) | 0-2  37.1(13/35) | AUC=0.89 | NA | For very poor outcome (90D mRS 5-6):  Infarct volume in brain stem: *P*<0.001; |
| Tong et al. 2020. [22]  (Futile recanalization) | Retrospective analysis on a prospective cohort  2012.01.2018.07 | BAO | ≤24 h | N=109 (SR) | 100% | 4-6: 44%(48/109) | 0.83 (0.75-0.90) | 4-items score  NIHSS: 0, 2  PMI: 0,1  Bilateral thalamic infarction: 0,1  BATMAN:0, 1 | NIHSS score(≥20 vs. ＜20): OR 5.65[2.13-14.99], *P*<0.01;  PMI(≥3 vs. ＜3): OR 2.27[0.84-6.13], *P*=0.09;  Bilateral thalamic infarction (yes vs. no): OR 3.67[0.83-21.42], *P*=0.08;  BATMAN collateral score (≤3 vs. ＞3 points): OR 2.92[1.15-7.41], *P*=0.02; |
| Pazuello, et al. 2021. [23] | Retrospective study  2011.06-2019.12 | BAO | ≤24 h | N=80 | 86.2% (69/80) | 0-2  26.2%(21/80) | NA | NA | Age: OR 0.9 [0.9-0.99], *P*=0.047;  Baseline NIHSS≤10: OR 2.90 [0.60-13.97], *P*=0.184;  Pc-ASPECT: OR 1.95 [0.98-3.88], *P*=0.054;  V4-proximal BA: OR 10.2 [1.45-72.15], *P*=0.019;  eTICI of 3: OR 4.67 [0.94-23.10], *P*=0.058; |
| Alexandre et al. 2021. [24] | Retrospective study  2016.01-2019.07 | BAO | ≤24 h | N=191 | 86.4%(165/191) | 0-2: 38.21%(73/191) | NA | NA | Baseline NIHSS: OR 0.77[0.61-0.96], *P*=0.025;  Pc-ASPECT: OR 3.01[1.03-8.76], *P*=0.043; |
| Kaneko et al. 2021. [25] | Retrospective study  2015.01-2019.03 | BAO | OTR: 285(217-538) | N=73 | 95.9%(70/73) | 0-2: 34.20%(25/73) | NA | NA | Age: OR 1.13[1.04-1.23], *P*=0.003;  NIHSS: OR 1.04[0.96-1.12], *P*=0.31;  Pc-ASPECT: OR 0.40[0.21-0.77], *P*=0.006;  Hemorrhage complication: OR 0.34[0.31-29.9], *P*=0.34;  OTR (min): OR 0.52[0.99-1.00], *P*=0.53;  mTICI: OR 0.11[0.03-1.43], *P*=0.11; |
| Sang et al. 2021. [26] | Retrospective study. BASILAR registry.  2014.01-2019.03 | BAO | ≤24 h | N=823  EVT=654  SMT=169 | 81.0%(530/654) | 0-2: 32.26%(211/654) | NA | NA | Baseline NIHSS: OR 0.92[0.90-0.94], *P*<0.001;  Baseline pc-ASPECT: OR 1.56[1.34-1.81], *P*<0.001;  DM: OR 0.57[0.33-0.99], *P*=0.045;  Distal BA: OR 2.41[1.30-4.47], *P*=0.005;  PTR: OR 0.94[0.90-0.97], *P*=0.001;  ASITN/SIR grade 0-1: OR 0.22[0.11-0.44], *P*<0.001;  ASITN/SIR grade 2: OR 0.37[0.19-0.73], *P*=0.004;  mTICI score 2b/3: OR 4.79[2.31-9.94], *P*<0.001; |
| Baik et al. 2022. [27] | Retrospective study  2008.03-2021.04 | ICPAO (P1\P2) | ≤24 h | N=48 | 68.8%(33/48) | 0-1: 52.1%(25/48) | NA | NA | Tax>6 s midbrain thalamus: OR 0.108[0.013-0.878], *P*=0.037; |
| Brooks et al. 2022. [28] | Retrospective study  2016.06-2019.06. | BAO | ＜24 h | N=118 | 71%(84/118) | 0-2: 44.9%(53/118) | 0.91 | NA | NIHSS: OR 0.90[0.84-0.90], *P*=0.006;  pcASCO: OR 1.91[1.25-2.92], *P*=0.003; |
| Park et al. 2022. [29] | Retrospective study.  2012.01-2019.11. | basilar top occlusion |  | N=42 | 78.6%(33/42) | 0-2: 50%(21/42) |  |  | Baseline NIHSS: OR 0.84[0.73-0.97], *P*=0.017;  Type III PCoA: OR 21.54[1.33-347.97], *P*=0.031; |
| Heit et al. 2023. [30] | Retrospective cohort study 2015.01-2019.12 | BAO | 4h (1:52-9:20) | N=102 | 84% (87/103) | ≥4  51% (53/103) | NA | NA | Age(per 5-year increment): OR 1.37 [1.08-1.72], *P*=0.008;  NIHSS: OR 1.11 [1.04-1.18], *P*=0.001;  SR(TICI 2b-3): OR 0.03 [0.003-0.25], *P*=0.002;  Pc-ASPECT≤6: OR 11.4 [1.73-75], *P*=0.011;  CAPS＞3: OR 26.22 [1.07-642], *P*=0.045; |
| Liu et al. 2023. [31] | Retrospective cohort study 2012.01-2018.12 | ABAO | OTP: 439.0(315.0-711.0) | N=116 | 82.8%(96/116) | 0-2: 44.0%(51/116) | NA | NA | Baseline NIHSS, per 1-point increase: OR 0.90[0.86-0.95], *P*=0.004;  IV-tPA: OR 5.27[1.38-24.39], *P*=0.002;  PMT score＜5: OR 14.29[5.73-35.67], *P*<0.001; |

ABAO, acute basilar artery occlusion; mRS indicates modified Rankin Scale; VBO, vertebrobasilar occlusion; AUC, area under curve; PC, posterior circulation; NIHSS indicates national Institute of Health Stroke Scale; mRS, modified Rankin scale; PC-ASPECT, posterior circulation Alberta Stroke Program Early CT Score; PMI, Pons-Midbrain Index; PCCS, the posterior circulation collateral score; BATMAN, the Basilar Artery on Computed Tomography Angiography; OTA, onset to admission time; OTP, onset to puncture time; PTR, puncture to reperfusion time; ASTN/SIR, America society of interventional and therapy neuroradiology/society of interventional radiology; NA, not available.

References in Supplementary materials

1. Singer, O.C., et al., *Mechanical recanalization in basilar artery occlusion: the ENDOSTROKE study.* Ann Neurol, 2015. **77**(3): p. 415-24.

2. Yoon, W., et al., *Predictors of Good Outcome After Stent-Retriever Thrombectomy in Acute Basilar Artery Occlusion.* Stroke, 2015. **46**(10): p. 2972-5.

3. Mokin, M., et al., *Clinical and Procedural Predictors of Outcomes From the Endovascular Treatment of Posterior Circulation Strokes.* Stroke, 2016. **47**(3): p. 782-8.

4. Bouslama, M., et al., *Predictors of Good Outcome After Endovascular Therapy for Vertebrobasilar Occlusion Stroke.* Stroke, 2017. **48**(12): p. 3252-3257.

5. Uno, J., et al., *Mechanical Thrombectomy for Acute Basilar Artery Occlusion in Early Therapeutic Time Window.* Cerebrovasc Dis, 2017. **44**(3-4): p. 217-224.

6. Giorgianni, A., et al., *Endovascular Treatment of Acute Basilar Artery Occlusion: Registro Endovascolare Lombardo Occlusione Basilar Artery (RELOBA) Study Group Experience.* J Stroke Cerebrovasc Dis, 2018. **27**(9): p. 2367-2374.

7. Li, C., et al., *Outcome of endovascular treatment for acute basilar artery occlusion in the modern era: a single institution experience.* Neuroradiology, 2018. **60**(6): p. 651-659.

8. Lin, S.F., et al., *Predicting functional outcomes of posterior circulation acute ischemic stroke in first 36 h of stroke onset.* J Neurol, 2018. **265**(4): p. 926-932.

9. Luo, G., et al., *Factors Associated with 90-Day Outcomes of Patients with Acute Posterior Circulation Stroke Treated By Mechanical Thrombectomy.* World Neurosurg, 2018. **109**: p. e318-e328.

10. Rentzos, A., et al., *Endovascular treatment of acute ischemic stroke in the posterior circulation.* Interv Neuroradiol, 2018. **24**(4): p. 405-411.

11. Yang, H., et al., *Early Diffusion-Weighted Imaging Brain Stem Score for Acute Basilar Artery Occlusion Treated with Mechanical Thrombectomy.* J Stroke Cerebrovasc Dis, 2018. **27**(10): p. 2822-2828.

12. Kang, D.H., et al., *Endovascular Thrombectomy for Acute Basilar Artery Occlusion: A Multicenter Retrospective Observational Study.* J Am Heart Assoc, 2018. **7**(14).

13. Ravindren, J., et al., *Predictors of Outcome After Endovascular Thrombectomy in Acute Basilar Artery Occlusion and the 6hr Time Window to Recanalization.* Front Neurol, 2019. **10**: p. 923.

14. Gramegna, L.L., et al., *Predictors of response to endovascular treatment of posterior circulation stroke.* Eur J Radiol, 2019. **116**: p. 219-224.

15. Kaneko, J., et al., *Endovascular treatment of acute basilar artery occlusion: Tama-REgistry of Acute Thrombectomy (TREAT) study.* J Neurol Sci, 2019. **401**: p. 29-33.

16. Zhang, X., et al., *Predictors of Good Outcome After Endovascular Treatment for Patients with Vertebrobasilar Artery Occlusion due to Intracranial Atherosclerotic Stenosis.* Clin Neuroradiol, 2019. **29**(4): p. 693-700.

17. Guenego, A., et al., *Neurological improvement predicts clinical outcome after acute basilar artery stroke thrombectomy.* Eur J Neurol, 2021. **28**(1): p. 117-123.

18. Jadhav, A.P., et al., *Predicting outcomes after acute reperfusion therapy for basilar artery occlusion.* Eur J Neurol, 2020. **27**(11): p. 2176-2184.

19. Kwak, H.S. and J.S. Park, *Mechanical Thrombectomy in Basilar Artery Occlusion: Clinical Outcomes Related to Posterior Circulation Collateral Score.* Stroke, 2020. **51**(7): p. 2045-2050.

20. Lee, S.J., et al., *Predicting Endovascular Treatment Outcomes in Acute Vertebrobasilar Artery Occlusion: A Model to Aid Patient Selection from the ASIAN KR Registry.* Radiology, 2020. **294**(3): p. 628-637.

21. Tajima, Y., et al., *Predictors of Very Poor Outcome after Mechanical Thrombectomy for Acute Basilar Artery Occlusion.* Neurol Med Chir (Tokyo), 2020. **60**(10): p. 507-513.

22. Tong, X., et al., *A Pre-Intervention 4-Item Scale for Predicting Poor Outcome Despite Successful Recanalization in Basilar Artery Occlusion.* Transl Stroke Res, 2020. **11**(6): p. 1306-1313.

23. Pazuello, G.B., et al., *Thrombectomy for Posterior Circulation Stroke: Predictors of Outcomes in a Brazilian Registry.* World Neurosurg, 2021. **147**: p. e363-e372.

24. Alexandre, A.M., et al., *Posterior Circulation Endovascular Thrombectomy for Large-Vessel Occlusion: Predictors of Favorable Clinical Outcome and Analysis of First-Pass Effect.* AJNR Am J Neuroradiol, 2021. **42**(5): p. 896-903.

25. Kaneko, J., et al., *Endovascular treatment of acute basilar artery occlusion: Outcomes, influencing factors and imaging characteristics from the Tama-REgistry of acute thrombectomy (TREAT) study.* J Clin Neurosci, 2021. **86**: p. 184-189.

26. Sang, H., et al., *Values of Baseline Posterior Circulation Acute Stroke Prognosis Early Computed Tomography Score for Treatment Decision of Acute Basilar Artery Occlusion.* Stroke, 2021. **52**(3): p. 811-820.

27. Baik, S.H., et al., *Mechanical thrombectomy for acute posterior cerebral artery stroke; Feasibility and predictors of outcome.* Neuroradiology, 2022. **64**(7): p. 1419-1427.

28. Broocks, G., et al., *New imaging score for outcome prediction in basilar artery occlusion stroke.* Eur Radiol, 2022. **32**(7): p. 4491-4499.

29. Park, J.S., et al., *Occlusion type and posterior communicating artery patency may predict favorable outcome after endovascular thrombectomy in selective basilar top occlusion.* Front Neurol, 2022. **13**: p. 1047971.

30. Heit., J.J., et al., *Imaging Factors Associated With Poor Outcome in Patients With Basilar Artery Occlusion Treated With Endovascular Thrombectomy.* Stroke: Vascular and Interventional Neurology, 2023.

31. Liu, L., et al., *Prognostic value of pretreatment diffusion-weighted imaging score for acute basilar artery occlusion with successful endovascular recanalization.* Neuroradiology, 2023. **65**(3): p. 619-627.
